# Predation and the Maintenance of Color Polymorphism in a Habitat Specialist Squamate

**DOI:** 10.1371/journal.pone.0030316

**Published:** 2012-01-25

**Authors:** Vincent R. Farallo, Michael R. J. Forstner

**Affiliations:** Biology Department, Texas State University-San Marcos, San Marcos, Texas, United States of America; University of Utah, United States of America

## Abstract

Multiple studies have addressed the mechanisms maintaining polymorphism within a population. However, several examples exist where species inhabiting diverse habitats exhibit local population-specific polymorphism. Numerous explanations have been proposed for the maintenance of geographic variation in color patterns. For example, spatial variation in patterns of selection or limited gene flow can cause entire populations to become fixed for a single morph, resulting in separate populations of the same species exhibiting separate and distinct color morphs. The mottled rock rattlesnake (*Crotalus lepidus lepidus*) is a montane species that exhibits among-population color polymorphism that correlates with substrate color. Habitat substrate in the eastern part of its range is composed primarily of light colored limestone and snakes have light dorsal coloration, whereas in the western region the substrate is primarily dark and snakes exhibit dark dorsal coloration. We hypothesized that predation on high contrast color and blotched patterns maintain these distinct color morphs. To test this we performed a predation experiment in the wild by deploying model snakes at 12 sites evenly distributed within each of the two regions where the different morphs are found. We employed a 2×2 factorial design that included two color and two blotched treatments. Our results showed that models contrasting with substrate coloration suffered significantly more avian attacks relative to models mimicking substrates. Predation attempts on blotched models were similar in each substrate type. These results support the hypothesis that color pattern is maintained by selective predation.

## Introduction

Understanding the mechanisms that generate and maintain biological diversity is a major goal of evolutionary biology. Geographic variation in one or more phenotypic traits has attracted the attention of evolutionary biologists because it is assumed that differences among populations represent adaptation to local environmental conditions and selective agents. Color polymorphism is a striking example of phenotypic biodiversity and has been documented in a variety of taxa [1], [2], [3], [4] prompting a basic question: Why have multiple phenotypes within a single species evolved and how are these phenotypes maintained? Whereas most recent interest has emphasized the mechanisms maintaining polymorphism within a population, e.g., density dependent selection [5], the factors responsible for structuring among population polymorphisms must include spatial variation. Thus, potential explanations include restricted gene flow among populations or morph specific predation. Heterogeneous habitats and substrates among populations have been shown to facilitate selection that contributes to color polymorphism [6], [7], [8], [9]. Furthermore, background matching, e.g., crypsis, may be important for evading detection by predators [10]. Geographic separation of a population combined with limited dispersal also reduces gene flow, which in turn favors natural selection for background matching and avoidance of predation. Most analyses of geographic variation in color patterns focus on prey species [7], [11]. For example, deer mice of the genus *Peromyscus* are one of the few color polymorphic vertebrates that have been studied extensively [12], [13], [14], [15]. Much work has gone into studying the genes that cause color polymorphism in this genus as well as the mechanisms that maintain the varying color morphs in nature. A recent study using plasticine models mimicking beach mice, *Peromyscus polionotus*, found that individuals that best match their background have a greater selective advantage due to reduced predation by visually orientated predators [9]. However, limited data are available for color polymorphisms in predator species while recent studies on snakes of the genus *Vipera* [16], [17], [18] represent an exception to this general trend.

There are three general hypotheses for the maintenance of color variation among populations; particularly in species that do not exhibit sexual dimorphism, i.e., both sexes have similar dorsal patterns. First, color variation may be related to thermoregulation. This mechanism has been supported by numerous studies focusing on vertebrates [19], [20] but has been demonstrated more commonly in insects and mollusks [21], [22]. Additional explanations are based largely on crypsis, which has been studied extensively [23], [24], [25]. The second hypothesis states that cryptic coloration is prevalent in a given area as a result of foraging success of cryptic predators. The third hypothesis proposes that cryptic coloration evolves within areas of different substrate type which effectively reduces potential predation from visually oriented predators. This hypothesis has been tested in several polymorphic taxa using a variety of methods (direct observation [26], model experiments [9], and virtual experiments [27]).

Mechanisms maintaining pattern polymorphism, e.g., blotching, stripes, or patternless, can be more obscure. In snakes, disruptive patterns such as banding or blotching are typically associated with cryptic or defensive behaviors, while striped or unpatterned individuals are associated with escape behaviors [28], [29]. This hypothesis has been tested and confirmed in several studies, which link changes in behavior with ontological changes in dorsal patterns [30], as well as linking increased survival rates for individuals where color pattern and escape behavior are positively correlated [31]. However, these studies focus on individuals within a population and do not address spatial variation in color polymorphism.

### Mottled rock rattlesnakes as a model system

Mottled rock rattlesnakes (*Crotalus lepidus lepidus*) of the arid southwest USA and northwest Mexico exhibit pronounced color and pattern polymorphisms throughout their geographic range. Current data suggest that these patterns are linked to background color-matching [32], [33]. Other species of rattlesnakes which are ostensibly monomorphic for color and pattern have been shown to exhibit background matching to substrate, which gives credence to the hypothesis that color morphs in rattlesnakes may be linked to crypsis [34]. However, to our knowledge, no study has directly addressed whether the color pattern of mottled rock rattlesnakes match surrounding substrates. We are also unaware of any previous studies that have addressed the maintenance of the color polymorphism found in *C. l. lepidus*. In addition, unlike prey species where predation is hypothesized to maintain color or pattern polymorphism, *C. l. lepidus* is a mesopredator that also has defensive weapons, i.e., venom with hemolytic enzymes in combination with neurotoxins, which is used for predator deterrence and prey subdual. Consequently, the maintenance of color polymorphism may represent a balance between prey acquisition and predator avoidance. Specifically, despite their defensive capabilities, rattlesnake species have many known visually-oriented predators including birds of prey (e.g., red-tailed hawks (*Buteo jamaicensis*), Swainson's hawks (*Buteo swainsoni*), and greater roadrunners (*Geococcyx californianus*) [33], [35], [36]).

**Figure 1:**
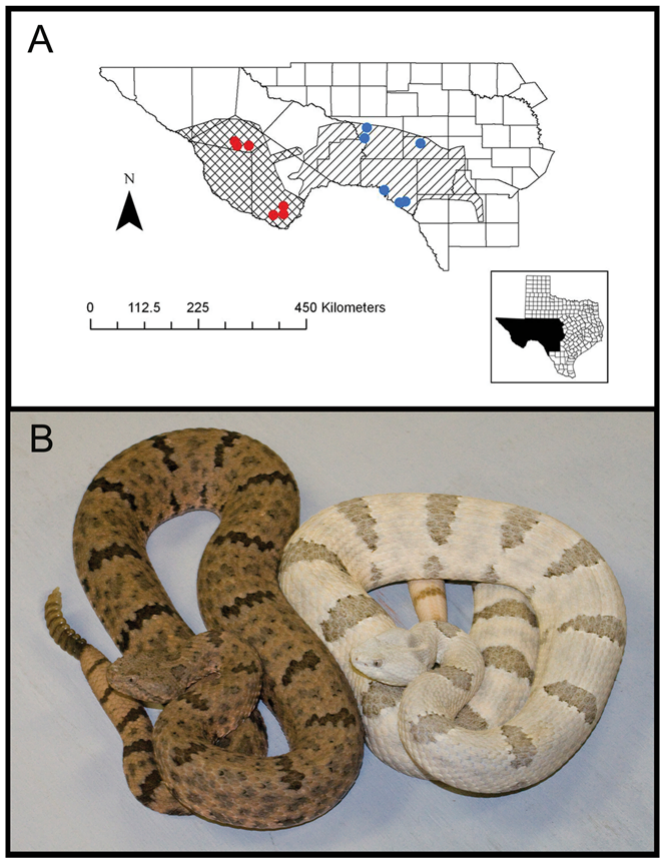
Modified range map of *Crotalus lepidus lepidus* and a photo of two specimens from the two races. (A) Map of southwestern Texas showing the ranges of the Stockton-Edward Plateau (Eastern) and Davis Mountains-Big Bend (Western) morphs described by Vincent (1982). The single hatched section represents the eastern morphs range in Texas and the double hatched section represents the western morphs range in Texas. Western field sites for the predation experiment are indicated by red dots and eastern field sites are indicated by blue dots. Note: this map does not illustrate the entire species range, but solely where the morphs were designated as being most prominent by Vincent (1982). (B) A side by side comparison of two live *Crotalus l. lepidus*. The dark colored snake on the left is from Brewster County in the western portion of their range. The light colored snake on the left is from Edwards County in the eastern portion of their range. Both specimens are part of the collection maintained by Michael Price at the San Angelo Nature Center, located in San Angelo, Texas.

*Crotalus l. lepidus* is found from southern New Mexico, through west Texas and into central Mexico [37]. Much of the species geographic distribution is composed of isolated populations inhabiting montane “islands” in “seas” of lowland desert [38]. Localized populations occupy two regions in west Texas (Figure 1A) that correspond with contrasting substrate type. These populations have been described as two distinct races based on differences in coloration and blotching frequency [20] (Figure 1B). Two morphs occur in eastern and western regions of west Texas [32]. Previously, Vincent [20] described localized “regions” in the distribution of *C. l. lepidus* within eastern and western divisions of west Texas where the color and pattern polymorphism are distinct. The two morphs of *C. l. lepidus* are distinguished by both color and blotch density (dorsal blotches contrasting with the predominant dorsal color). The eastern morph, located in the Stockton and Edwards plateaus, is characterized by blue- and light-gray coloration. Individuals in this region occur on light colored soil or limestone outcrops [32]. Moreover, this morph has anterior fading of blotches resulting in a uniform unblotched pattern on the dorsal-anterior section of its body. The western morph ranges from the Davis Mountains westward and southward through the Big Bend region, exhibits more color variation with typically pink or buff base coloration, and inhabits more variable substrates comprised of darker soils and volcanic rocks. The western morph has a higher blotch density resulting in more distinct and consistently darker pattern than the eastern morph [32]. However, the differences in color pattern are typically more distinct between regions, whereas the extent of the differences in blotching is not always as striking (e.g., Figure 1B).

More recent surveys found that light and dark morphs occur throughout west Texas, which led Forstner et al. [39] to suggest that the frequency of morphs correlates with substrate type. The spatial correlation between substrate type and color morph raises the question of what maintains the polymorphism. Similar species found throughout the same region, e.g., *Crotalus atrox*, lack the spatial pattern in dorsal coloration. Differences in dispersal capacities and habitat requirements have been proposed as hypotheses explaining the absence of color variation in some species. Greater dispersal capacities and generalized habitat preferences could potentially favor more homogenous colors and patterns. In contrast, *C. l. lepidus* is restricted to rocky substrates, which may favor unique color and pattern types for a given area. Consequently, we hypothesize that variation in predation risk associated with different habitat types maintains the color polymorphism observed in *C. l. lepidus*. This species provides an excellent opportunity to investigate the role of predation in maintaining color and pattern polymorphisms of a higher trophic level vertebrate taxon.

In this study, we first tested the hypothesis that mottled rock rattlesnakes of varying color morphs are more cryptic on their local substrates than they are on substrates where different color morphs are found. We used reflectance data of mottled rock rattlesnakes from light and dark color morphs and compared these values to reflectance data from different rocks throughout the snakes range. Second, based on the distribution of color morphs in *C. l. lepidus* we tested the hypothesis that spatial variation in natural selection maintained the polymorphism among populations. We hypothesized that morphs with a color pattern that contrasted with the local substrate type had an increased risk of predation. A hypothesis of spatial variation in mortality predicts that light morphs should have higher survivorship in localities with light substrates and dark morphs prevail on dark substrates. We tested this hypothesis in a predation experiment using model snakes that varied in color patterns. Models are commonly used in predation studies [40], [41], [42], because they provide the opportunity to manipulate color patterns while controlling for other traits, e.g., posture, body size. Multiple studies have employed model snakes to investigate the covariation between predation risk and color pattern [18], [41]. In this study, we deployed models of *C. l. lepidus* across localities in west Texas that varied in color pattern and dorsal blotching to estimate the risk of predation on different substrate types. Our results suggest cryptic individuals have a survival advantage compared to individuals that contrast with substrate color. Consequently, we conclude that spatial variation in natural selection maintains the color morphs of this species.

## Materials and Methods

### Ethics statement

When required, we obtained permission to utilize all field sites from owners or managers. Permit numbers are included when applicable. Big Bend National Park, Raymond Skiles, permit #: BIBE-2009-SCI-0029; Amistad National Recreation Area, Greg Garetz, permit #: AMIS-2009-SCI-0003; all right-of-way field sites, Cal Newman and Christopher Maldonado, permit #: SPR-0102-191; Chihuahuan Desert Research Institute, Cathryn Hoyt; Fort Davis private property, James R. Dixon; and Seminole Canyon State Park and Historic Site, Randy Rosales. Reflectance measurements of mottled rock rattlesnakes were carried out in strict accordance with the recommendations in the Guide for the Care and Use of Laboratory Animals of the National Institutes of Health. The protocol was approved by the Institutional Animal Care and Use Committee of Texas State University-San Marcos (Permit #: 1010_0501_09).

### Model construction

**Figure 2:**
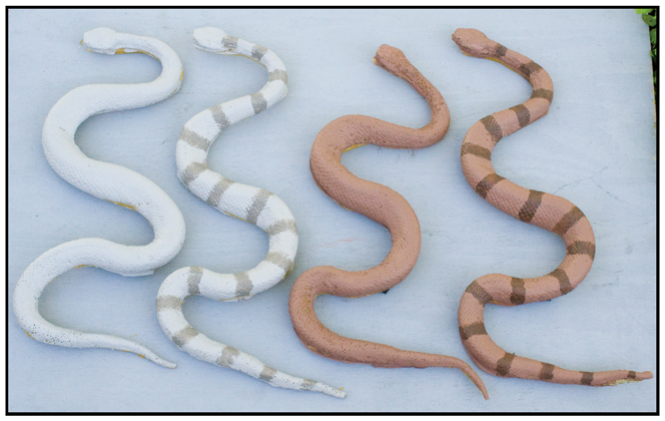
Photograph of the four treatments of model snakes. The treatments represent the phenotypic extremes of *Crotalus l. lepidus* found in eastern and western regions of west Texas (refer to Figure 1A). The treatments are (from left to right) eastern unblotched, eastern blotched, western unblotched, and western blotched.

We constructed models using urethane foam rather than plasticine. The former is an ideal model material because it retains predation marks, yet can withstand temperatures in excess of 38°C characteristic of west Texas environments. We constructed molds using Reoflex 40 urethane rubber (Smooth-On; Easton, PA) and a deceased adult broad banded copperhead (*Agkistrodon contortrix laticinctus*), to mimic a typical Crotalid body shape. Foam iT! 3 urethane foam (Smooth-On; Easton, PA) was poured into the molds and allowed to set for two hours. Models were painted using acrylic paint to represent two variations of the species natural color combined with two pattern types (unblotched or completely blotched) to represent relative phenotypic extremes within this species (Figure 2). Color of models was based on photos of multiple specimens from known localities and substrate types, specifically from within the eastern region dominated by limestone substrate and the western region dominated by volcanic rock substrate. Blotch numbers per snake was taken from Vincent [32] who found an average blotch number of 18.9 for the western region and 17.7 for the eastern region of *C. l. lepidus*. We used an average of the two for 18 total blotches on all blotched models.

### Field sites

We placed snake models in six eastern and six western regions of the range of *C. l. lepidus* in Texas (Figure 1A). Furthermore, we split the eastern and western regions into northern and southern sections. Each section contained three separate sites. A minimum of 10 km and a maximum of 100 km separated the field sites within each section while a minimum of 113 km and a maximum of 333 km separated different sections. This design allowed us to test for differences in estimated predation rates of the eastern and western regions which have contrasting phenotypes, as well as northern and southern sections within each region which do not have the contrasting phenotypes of *C. l. lepidus*. The distribution of field sites throughout each region was intended to provide insight into the frequency of predation occurring on differing phenotypes over a large portion of the species range. Finally, the number of individual field sites allowed us to put adequate distance between the placements of models to avoid one model from influencing the detectability of another model while also ensuring we avoided placing models in areas that would have been excessively dangerous for us to work.

### Quantifying avian perception of mottled rock rattlesnakes and our models

An Ocean Optics Jaz UV/Vis Spectrophotometer (Model EL 200) with included Jaz-PX Xeon light source with a QR400-ANGLE-UV reflectance probe was used to measure percent reflectance of the model snake base colors, limestone and volcanic rocks from the eastern and western region, respectively, and mottled rock rattlesnakes from various locations throughout their range. Reflectance measurements were taken for 17 rocks from seven different sites, three within the western range (11 total volcanic rocks) and four within the eastern range (6 total limestone rocks). We took reflectance measurements for eight snakes from the San Angelo Nature Center, which houses a large collection of rock rattlesnakes from known localities. We measured three snakes from the eastern region found in areas of limestone (East-Limestone), three from the western region found in areas of volcanic rock (West-Volcanic), and two snakes from outside our study regions in the far western portion of the *C. l. lepidus*'s range found in areas of limestone (West-Limestone). The reflectance probe was held in a Mikopark CSH-45° holder to ensure all readings were taken at the same distance. All measurements were taken relative to a 99% Spectralon diffuse reflectance standard (SRS-99) using SpectraSuite software (Ocean Optics; Ver. 2.0.154). To reduce noise, the average of 10 reflectance scans with a boxcar width of five was used for each measurement. Additionally, the mean reflectance value from measurements taken at a minimum of three locations on each sample was used for all analyses.

In order to analyze the reflectance data in a way that would be consistent with the capabilities of avian vision we implemented Osorio and Vorobyev's physiological model [43] which considers the chromatic and achromatic contrast between colors as perceived by an animal with a tetrachromatic visual system. This analysis integrates cone sensitivity curves which provide the sensitivities to wavelengths for each of the four cones that birds possess, and also considers chromatic and achromatic properties as independent sources of visual information, as suggested from avian studies [44]. Achromatic contrast is especially important because it is crucial for distinguishing between small objects (or objects that appear small over large distances) [44]. Avian predators would most likely be detecting the model snakes mid-flight or while scanning over large open areas making achromatic contrast critical for model detection. We also incorporate chromatic contrasts because more refined detection may also be utilized by avian predators, especially by the primarily terrestrial greater roadrunner. The model measures the chromatic and achromatic contrast between color signals accounting for photoreceptor and background noise, and is expressed in just noticeable differences (JND), where a JND value of 1 is considered the threshold value for discrimination between two colors. For our analysis we utilized the “V” type cone sensitivity curve (the prevalent visual system found in birds of prey and other avian predators) of Endler and Mielke [45], using the software AVICOL [46]. Achromatic contrast was calculated from the sum of the spectra of MWS and LWS cones. Reflectance curves were smoothed using local and triangular smoothing options in AVICOL prior to all analyses.

### Predation experiment

We placed 40 models in each of the twelve sites to test the prediction that selective predation maintains the color polymorphism in snakes. We had 10 replicates of each combination of color and blotching per site ( = 480 total models). Models were left in place for a period of 14 d. We secured individual models to rocks with adhesive backed Velcro® at approximately 10 m intervals within typical *C. l. lepidus* habitat. Where possible, models were placed along a single linear transect (all following the edge of a cliff); and multiple transects were used to accommodate all 40 models when a single linear transect was not possible (multiple transects stacked above the cliff edge). Models were randomly placed on rocks with the lowest amount of overhead obstruction (vegetation, large rock formations, etc.) as possible.

Experiments were initiated during two time intervals, between 13-May-2009 and 18-May-2009 and then between 4-June-2009 and 10-June-2009. The first placement included all northern sites in both the eastern and western region while the second placement included all the southern sites. This was done to reduce the chance of extremes in weather influencing predator behavior more heavily in one region.

We photographed models at the time of deployment and after 14 d using a Canon XT digital SLR camera. Damage to models was scored as breaks, pecks, bites, or unknown origin. Avian attacks could be identified by breaks or pecks with triangle or circular punctures. Non-predator disturbances included damage to models that included bites from rodents and ungulates and unidentifiable marks.

Birds tend to attack the head of prey items [47], [48]; therefore we quantified the location of damage to the models to determine if models incurred more damage in the head region for putative avian attacks. We measured the distance from the anterior end of the model to each predation mark on the model. In the case of a single mark on a model we measured from the tip of the head to the predation mark. In the case of multiple marks on a model, we made the same measurement for each mark and then averaged the distances. We also recorded the total length of each model for use as a covariate due to the few instances of tail breaks that had occurred pre-deployment and moderate shrinkage that occurred following molding.

### Statistical analysis

The AVICOL analysis generated comparisons between all possible combinations of models, rocks, and snakes. The JND values for achromatic and chromatic contrast were not normally distributed even after multiple attempts at transformation to conform to normality; therefore we used a permutation ( = 1000) based ANOVA for all analyses based on response variables derived from the achromatic and chromatic JND values. Comparisons of achromatic and chromatic contrasts between model snakes and rocks were conducted using a 2×2 factorial permutation ANOVAs. Both the model snake and rock factors had two varieties, designated as Eastern and Western, which is based on the regions the different snake morphs that the models were mimicking and the location where the rocks were found. A 3×2 factorial permutation ANOVA was used for the comparison between mottled rock rattlesnakes and rocks. For the analysis with mottled rock rattlesnakes as a factor, three varieties were used, designated as East-Limestone, West-Volcanic, and West-Limestone. The snakes found on limestone from the western portion of the species range were separated from the eastern region snakes despite a similar appearance because they were from an area not included in our predation study and are in closer proximity to areas of darker substrates that dominate much of the western portion of the *C. l. lepidus*'s range. However, we included the West-Limestone snakes as further evidence of background color-matching. If an analysis was significant we performed post-hoc individual one-way permutation ANOVAs to determine which factors were significantly different. A Bonferroni correction was applied to all post-hoc analyses reducing our significance threshold of α = 0.05 to α = 0.025. All permutation ANOVA analyses were conducted using the lmPerm package [49] for the statistical program, R [50].

Region, section, site, color, and blotching type were the predictor variables used in the predation experiment analysis. Site refers to individual field sites and was nested within section and region. Section refers to the four major areas in which field sites were located (a northern and southern area within the eastern and western regions) and was nested within region. Both color and blotching were crossed together as well as being crossed with region. Attack and non-predator disturbance data were analyzed with a generalized linear model corrected for overdispersion under a Poisson distribution with a log link function with number of avian attacks and non-predator disturbances as the response variables [51]. The Poisson distribution is ideal for analyzing count data used in this predation study. The full model was analyzed separately for both avian attacks and non-predator disturbances. We started with the full model and sequentially removed terms until all combinations had been analyzed. In order to find the most parsimonious model with the highest support we utilized Akaike's information criterion corrected for small sample size (AIC_c_), evidence ratios, and AIC_c_ weights (*w_i_*) which were calculated for all significant models [52]. The effect tests generated from the generalized linear model analysis were used to interpret which parameters significantly affected the number of avian attacks or non-predator disturbances.

A separate analysis of damage location used an ANCOVA comparing distance of damage from the head as the response variable and type of damage (avian attack or non-predator disturbance) as the independent variable with the total length of the model as a covariate. All analyses were done using JMP (Version 7.0.1. SAS Institute Inc., Cary, NC, 1989–2007) unless indicated otherwise. All 40 models at the Burro Mesa site, located in Big Bend National Park (BBNP), were damaged. This damage was presumably caused by arthropods because all models incurred similar damage which consisted of small bites encompassing the entire dorsal side of each model. Therefore, we excluded data from this site during all analyses. Additionally, 19 missing models at the Point of Rocks site appeared to indicate human interference and were also excluded from all analyses.

## Results

**Figure 3:**
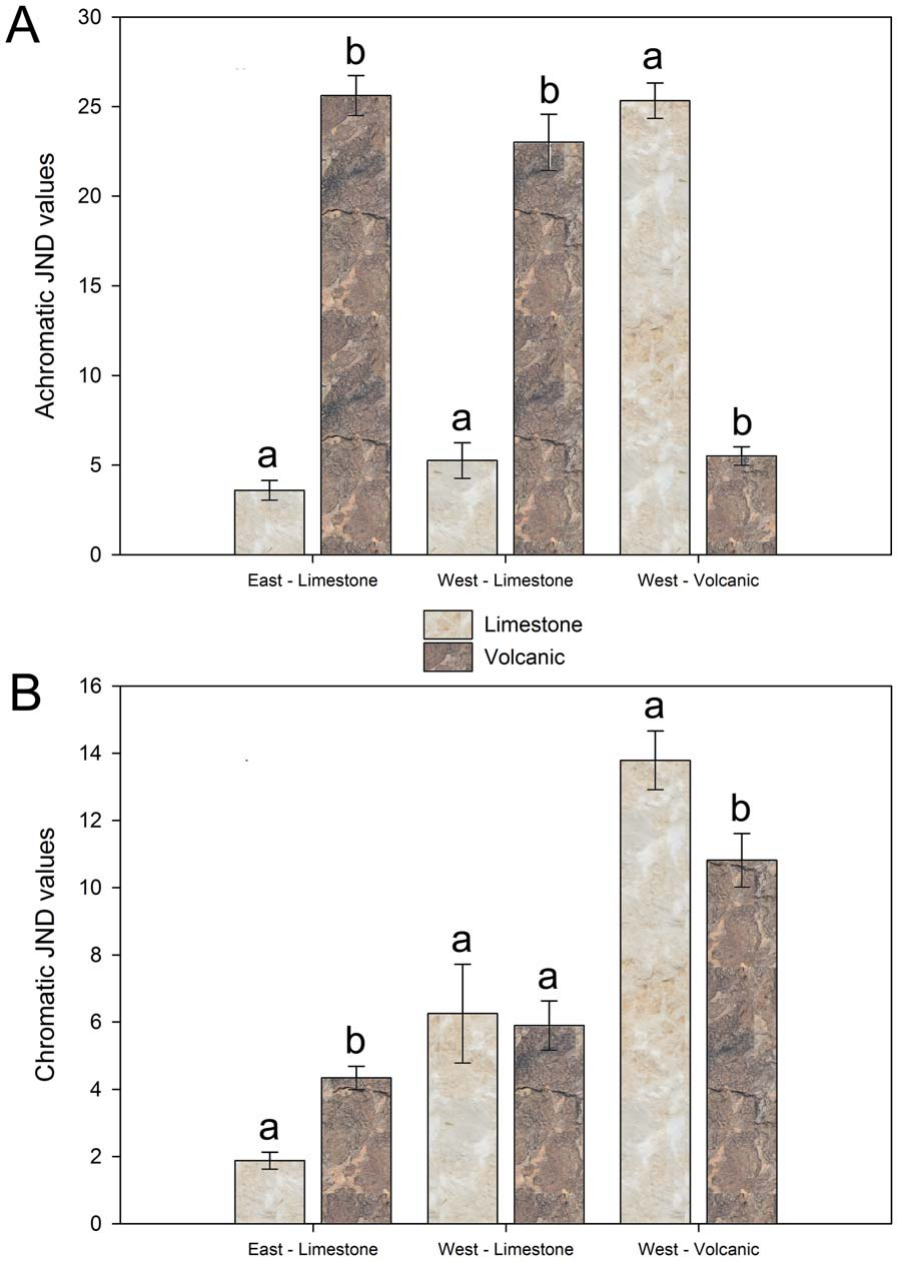
Mean achromatic and chromatic contrast values for mottled rock rattlesnake morphs on limestone and volcanic rock. Contrast values given in just noticeable differences (JND) for comparisons of limestone and volcanic rocks to three morphs of mottled rock rattlesnakes. The upper graph (A) shows achromatic contrast while the lower graph (B) shows chromatic contrast. The three morphs of snakes used are listed on the z-axis. East-Limestone includes three snakes found in the eastern region on limestone (see Figure 1). West-Limestone includes two snakes found outside of the study region used in this study, found in northern Hudspeth and Culberson counties (Texas) in the very western range of *C. l. lepidus*, unlike most areas from this section of their range, these individuals were found in areas with limestone substrate. Finally, West-Volcanic refers to three snakes found in the western region on volcanic rock (see Figure 1). Different letters above bars indicate significant differences between contrast values for limestone and volcanic rock for each snake morph. If the letter above the limestone and volcanic rock bars are identical for a snake morph, there is no significant difference in the contrast level for that snake morph between limestone and volcanic rock.

**Figure 4:**
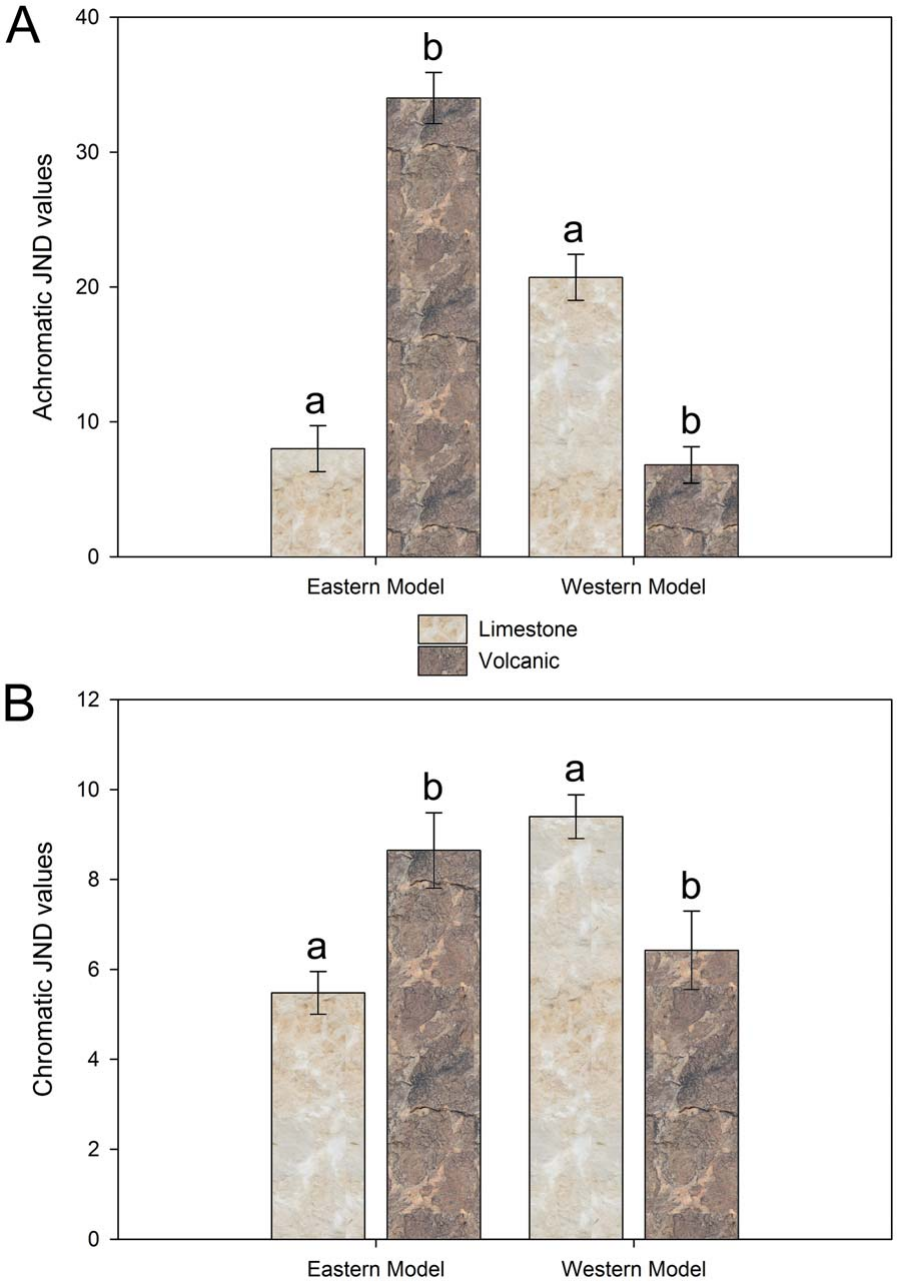
Mean achromatic and chromatic contrast values for model snake used in on predation study on limestone and volcanic rock. Contrast values given in just noticeable differences (JND) for comparisons of limestone and volcanic rocks to the two base model colors of our study (see Figure 2). Model type is listed on the x-axis with Eastern Model referring to the model color mimicking snakes from the eastern region in areas of limestone, while Western Model refers to the model color mimicking snakes from the western region on volcanic rock. Different letters above bars indicate significant differences between contrast values for limestone and volcanic rock for the individual model types.

We found significant differences between the achromatic and chromatic contrasts for the combinations of the three varieties of mottled rock rattlesnake and types of rock (Figure 3; achromatic: *F*_1,30_ = 118.124, *P*<0.0001; chromatic: *F*_1,30_ = 12.538, *P* = 0.001). There was also a significant difference between the achromatic and chromatic contrasts calculated for the two varieties of models and the two varieties of rocks (Figure 4; achromatic: *F*_2,130_ = 236.785, *P*<0.0001; chromatic: *F*_2,130_ = 7.055, *P* = 0.001). All post-hoc analyses (note: α = 0.025 as per Bonferroni correction for all post-hoc analyses) compared a snake morph or model type to limestone and volcanic rock. East Region-Limestone snakes had significantly greater achromatic and chromatic contrast on the volcanic rocks (achromatic: *F*_1,49_ = 197.85, *P*<0.0001; chromatic: *F*_1,49_ = 23.566, *P*<0.0001), Western Range-Limestone snakes only had significantly greater achromatic contrasts on volcanic rocks (achromatic: *F*_1,49_ = 62.247, *P*<0.0001; chromatic: *F*_1,49_ = 0.060, *P* = 0.809), and the West Region-Volcanic snakes had significantly greater achromatic and chromatic contrasts on the limestone rocks (achromatic: *F*_1,49_ = 383.64, *P*<0.0001; chromatic: *F*_1,49_ = 5.591, *P* = 0.022). The model snakes mimicking the snakes found on limestone had significantly greater achromatic and chromatic contrasts on volcanic rocks (achromatic: *F*_1,15_ = 80.13, *P*<0.0001; chromatic: *F*_1,15_ = 6.930, *P* = 0.019), while model snakes representing the snakes found on volcanic rock only had significantly greater achromatic contrasts on limestone, however chromatic contrasts was nearing significance (achromatic: *F*_1,15_ = 38.33, *P*<0.0001; chromatic: *F*_1,15_ = 5.669, *P* = 0.031). These results support the hypothesis that snake morphs are more cryptic on substrates on which they are typically found. Critical to the interpretation of our predation experiment, results also confirm that our models, representing the different snake morphs, exhibit similar levels of contrasts on the different substrates as did actual mottled rock rattlesnakes. Therefore, we are able to interpret our model snake predation study results accordingly.

**Figure 5:**
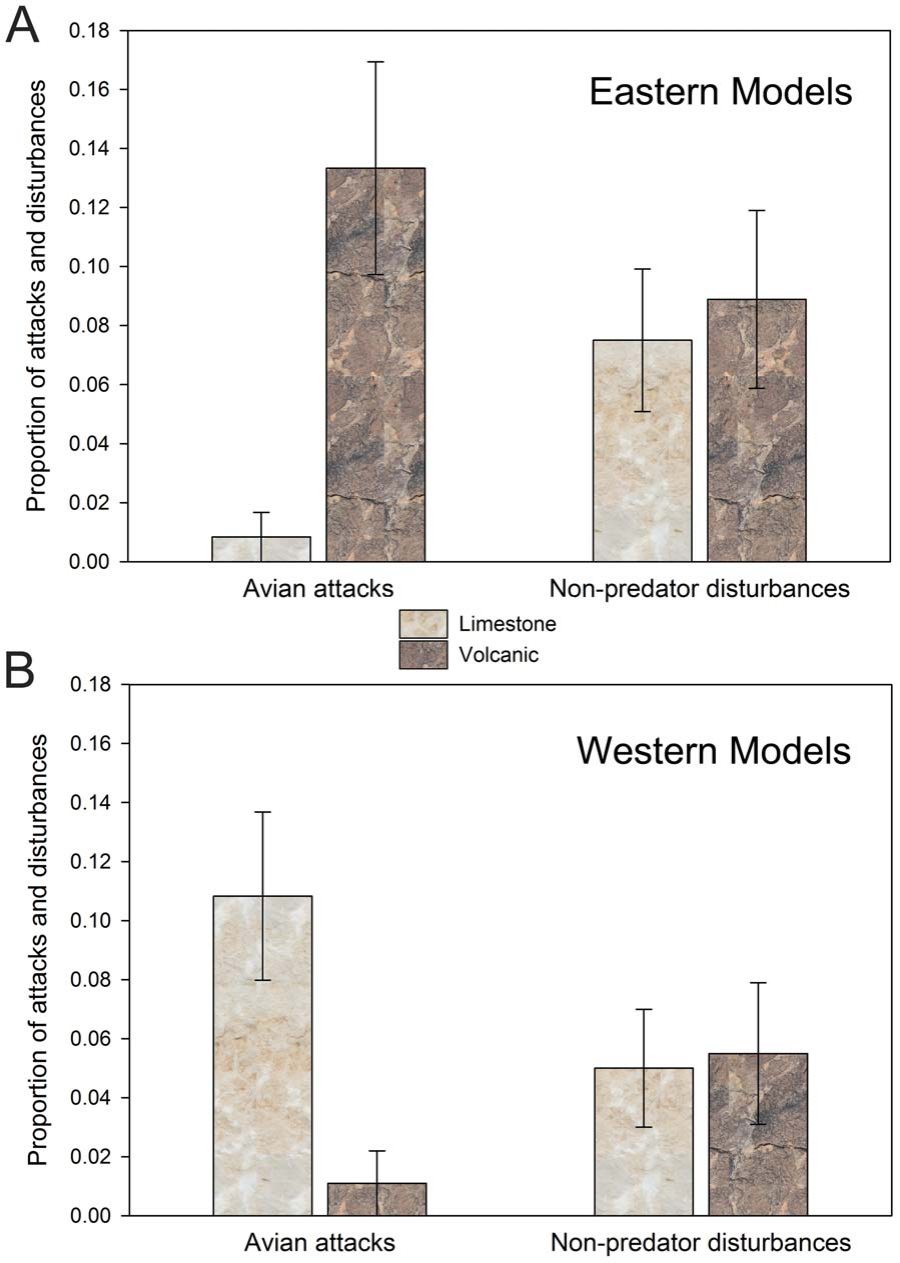
Proportion of avian attacks and non-predator disturbances of artificial snake models. Models were placed in the eastern region on limestone rocks and western region on volcanic rocks (refer to Figure 1A) during the course of the predation study. The upper graph (A) is showing the proportion of attacks on models that mimicked the coloration of *Crotalus l. lepidus* from the eastern region. The lower graph (B) is showing the proportion of attacks on models that mimicked the coloration of *C. l. lepidus* from the western region. The x-axis indicates the type of damage the models sustained as either avian attacks or non-predator disturbances.

**Figure 6:**
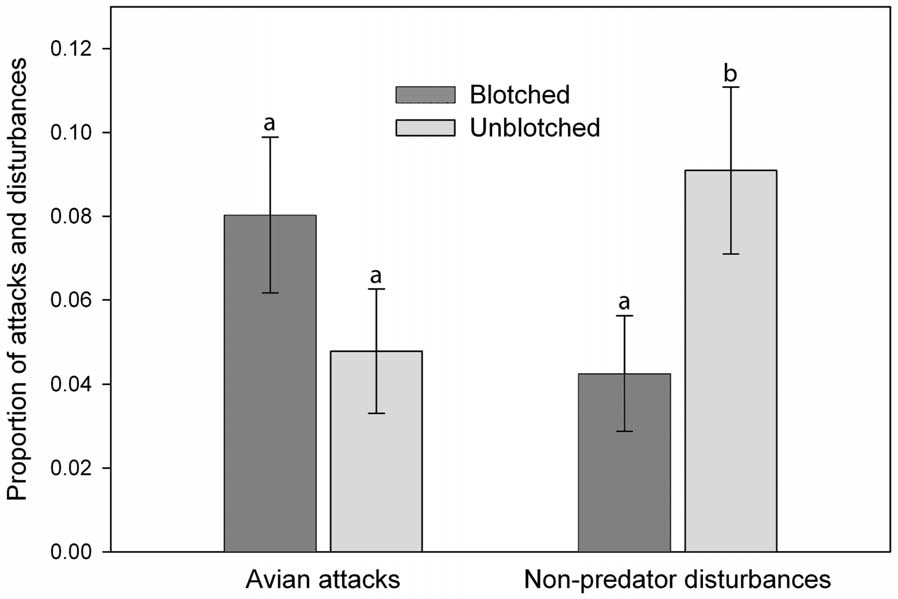
Proportion of avian attacks and non-predator disturbances on blotched and unblotched models. Different letters above bars indicate significant differences between the proportion of avian attacks or non-predator disturbances of blotched and unblotched models.

There were 27 avian attacks and 28 non-predator disturbances on 421 models over the 14 days of exposure. This equates to 13% of all models being attacked by avian predators (6%) or disturbed by non-predators (7%). Thirteen of the statistical models with avian attacks as the response variable were significant (*P*<0.05), and all of these models contained the region crossed with color parameter (Table 1). In addition, the model containing color, region, and color crossed with region best fit the data as determined using AIC_c_, and color crossed with region was the only significant parameter (χ^2^ = 24.62, *df* = 1, *P*<0.001). More avian attacks occurred on models whose dorsal coloration contrasted with the substrate (Table 1; Figure 5). None of the selected models for either avian attacks or non-predator disturbances included any interaction between blotching and region (Table 1). Only three of the statistical models with non-predator disturbances as the response variable were significant. The best model only contained the blotching parameter (χ^2^ = 4.05, *df* = 1, *P* = 0.04) indicating that non-predator disturbances occurred on non-blotched models more frequently (Figure 6), and that there was no interaction between the blotching type and region (Table 1).

**Table 1:** Model selection results from the avian attack and non-predator disturbance analysis.

Model	K	LL	AIC_c_	Evidence ratio	AIC_c_ *w_i_*
Avian Attacks					
Color+Blotching+Region+Region*Color+Region*Blotching	7	13.735	28.006	14.731	0.041
Color+Blotching+Color*Blotching+Region+Region*Color	7	12.859	27.131	9.509	0.063
Color+Region+Section[Region]+Region*Color	6	14.192	26.395	6.582	0.091
Color+Blotching+Region+Region*Color	6	12.680	24.883	3.091	0.193
Color+Region+Region*Color	5	12.482	22.626	1.000	0.597
Non-predator Disturbances					
Blotching	3	2.023	8.081	1.000	0.999

Only the significant models (*P*<0.05) which demonstrated some level of potential importance according to the number of parameters (K), log likelihood (LL), Akaiki Information Criteria corrected for small sample size (AIC_c_), evidence ratio, and AIC_c_ weight (*w_i_*) are presented. In the column presenting the models an asterisk (*) indicates that parameters are crossed and brackets ([…]) indicate that the preceding parameter is nested within the bracketed parameter. The model containing Color+Region+Region*Color appears to be favored for avian attacks, while the model containing Blotching is favored for non-predator disturbances.

**Figure 7:**
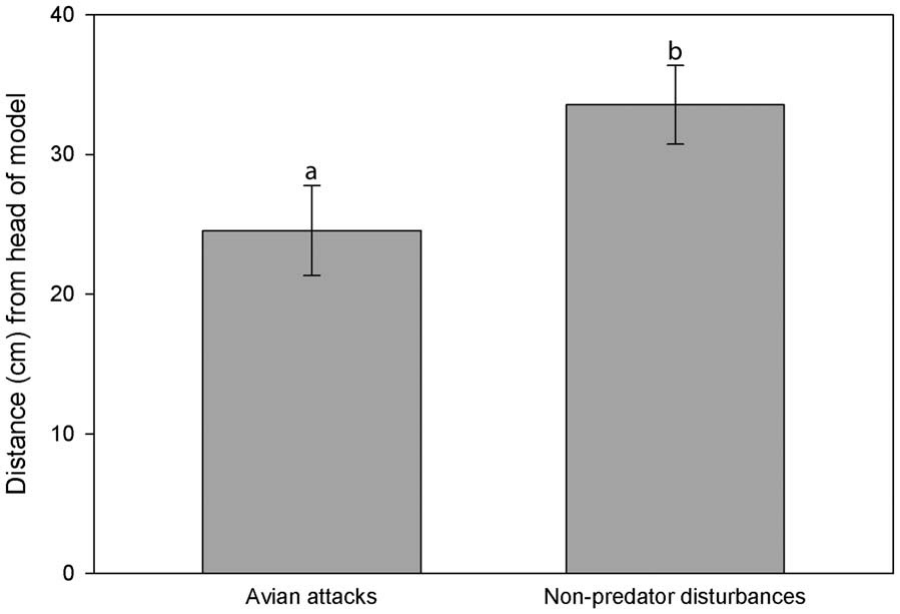
Location on models where damage caused by predators and non-predators occurred. Mean distance from head for damage caused by predators was 24.54 cm and 33.54 cm for non-predators. Different letters above bars indicate significant differences.

All 27 avian attacks were used in the analysis of damage location. We excluded four of the 28 total non-predator disturbances, because excessive damage to the models made it difficult to determine the exact location of all damage marks. Models that were determined to be attacked by avian predators had damage occur significantly closer to the head than models damaged by non-predators (*F*_1,48_ = 3.13, *P* = 0.05; Figure 7).

## Discussion

Results from our analyses indicate that individuals of *C. l. lepidus* that closely match the surrounding substrate have significantly lower rates of predation attempts than contrasting models. This supports the hypothesis that spatial variation in selective predation maintains the observed color polymorphism. However, these results do not refute the alternative hypotheses, but do indicate that selective predation likely plays a key role in the maintenance of color polymorphism in this species, potentially in conjunction with other mechanisms. In contrast, there was not a significant difference in the number of avian attacks on the different blotching treatments; however unblotched models were damaged by non-predator disturbers more often, regardless of the region in which the models were placed.

**Figure 8:**
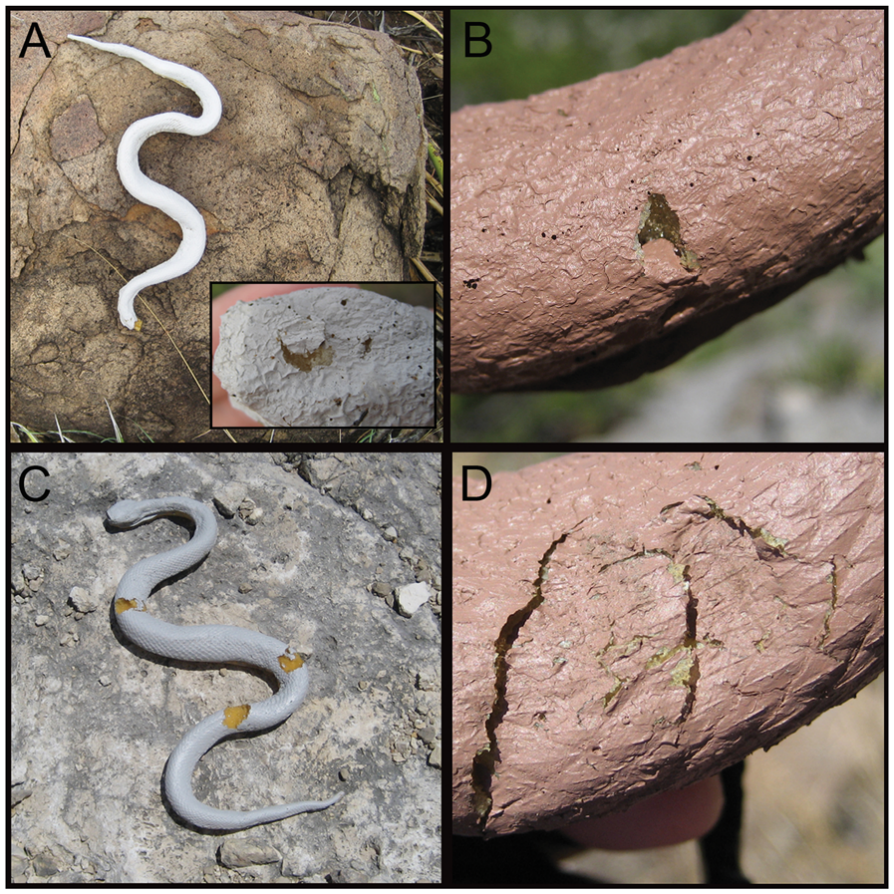
Examples of damage to model *Crotalus l. lepidus* during the course of the model predation study. All photos show damage on the dorsal surface of models. (A) An example of a decapitated model, note the decapitated head in the bottom right hand corner which has a single peck mark in the center. Typically models found with breaks were either decapitated or broken in several locations. (B) An example of a model showing a peck mark. Peck marks include single circular and triangular marks such as the one shown in this photo and also pairs of triangle shaped pecks indicating an open beak. (C) An example of a model showing bite marks. Bites were either full round chunks removed from the model or the impression of teeth marks left in the model. (D) An example of a model showing a questionable mark. In this case it is clear that a Barbary sheep, *Ammotragus lervia*, had stepped on the model. However, other questionable attacks include potential arthropod damage and incidences where it was unclear if an avian or non-predator attack occurred.

A main caveat of predation studies using models is the level of confidence in distinguishing between predation attempts and non-predator disturbances. Puncture marks left in models were similar to marks made on clay models in other artificial snake studies such as triangle, “U”, and circular shaped puncture marks [41], [53]. This type of damage has been routinely associated with avian attack. We also included models which were broken and showed no signs of non-predator disturbances such as bite marks (Figure 8). Non-predator disturbances were less obvious but it was apparent that some damage to models was caused by Barbary sheep, *Ammotragus lervia*, which had created hoof imprints in the foam. Some bite marks were chisel shaped that correspond with rodent teeth. All other damage that could not be confidently tied to a predation attempt was considered a disturbance. In total, 6% of the models were damaged by avian predators while 7% were damaged by non-predator disturbers during our experiment, which is similar to previous studies utilizing artificial organisms to record predation [41], [54]. The analysis of damage location indicated that marks noted as avian attacks were significantly closer to the head than marks made by non-predator disturbers. This result supports the distinction between predators and non-predator disturbers, as predators would be more likely to attack the head region in an attempt to kill the snake, whereas non-predators would make marks randomly.

The majority of studies concerning evolutionary interactions of non-sexual behavior and color polymorphism emphasize invertebrates [21], [24], [55] with a smaller number of studies based on vertebrates [31], [56], [57]. Varying dorsal patterns have been shown to coincide with different escape mechanisms such as in garter snakes (*Thamnophis ordinoides*) which cause optical illusions when undergoing certain movements aiding in escape potential [31]. Additionally, juvenile racers, *Coluber constrictor*, have blotched dorsal patterns, while adults have very uniform patterns. Creer [30] showed that juvenile racers were more likely to defend against predators while the uniform patterned adults were more likely to flee. Two color morphs of red-backed salamanders, *Plethodon cinereus*, were shown to exhibit different behaviors in the presence of predators [57]; however, it is not clear if the behavior or the color pattern have directly or indirectly caused the evolution of the other. The heterogeneity of the environment may also magnify the level of crypsis [27] depending on an individual's position within the environment. Behavioral and ecological factors need to be considered further when addressing questions of color polymorphism rooted in selective predation.

The absence of differing attack rates on the blotching treatments suggests alternative mechanisms. Given the sizeable difference between predation rates of the models mimicking the color of the region in which they were placed compared to the predation rates of models mimicking the color of the opposite region, it would appear likely that if selective predation was a cause of the different blotching patterns in the two regions that some differences would have been observed, especially given the extremes in blotching pattern which were used. However, in our study, the models were placed in open areas for the entire 14 d period. We did not mimic the activity patterns of the snakes, which would include a period of inactivity. Moreover, we could not take into account varying behaviors which may only expose snakes to predators for short periods of time during specific times of the day. Therefore, our study suggests that if differences in blotching patterns are a product of selective predation, then this is rooted in differential behaviors or activity levels in the contrasting habitats. Furthermore, unblotched models were damaged by non-predator disturbers more often which could be a function of the stationary nature of the models. Although other studies have typically found that blotched snakes are cryptic when stationary [30], [31], these studies have not taken into account the type of substrate that the snakes typically use, nor do they consider the time of day that predators are more likely to attack the snakes used in their studies. Blotching may help conceal a snake while moving when the blotches are not highly contrasting with the snake's body color, the substrate in the habitat is very heterogeneous, or if predators attack during periods of low light. However, all our models were stationary throughout the study, in many cases placed on open rock, which may have made the unblotched models less conspicuous and therefore more likely to be accidently disturbed by an ungulate walking through the study site. Additionally, although there was not a statistically significant difference between blotched and patternless treatments, more avian attacks occurred on blotched models (17) than on unblotched models (10), which would also support the hypothesis that, when stationary, the blotched models are more conspicuous. The correlation of differing behaviors and selective predation has been suggested in other taxa but primarily in experiments focusing on varied micro-habitat selection particularly between sexes rather than large scale habitat difference over a species range [58]. Additionally, different habitats, even in close proximity, can affect aspects of the ecology of *C. l. lepidus*. The work by Beaupre [59], [60], [61] demonstrated that energy budgets and activity levels of *C. l. lepidus* differed at two sites within Big Bend National Park. Notably, Beaupre's sites represented habitat found in both the western and eastern regions. This result suggests that among population variation in color morphs may be maintained by correlational selection operating on behavior and dorsal coloration (blotching). Future studies examining more comprehensive aspects of species ecology might be useful in making more robust correlations between snake color and pattern polymorphism, and their connection to differential behaviors.

Our study represents one of very few examples of selective predation maintaining color polymorphism in vertebrate taxa from a high trophic level, specifically among-populations. Although our study was based on *C. l. lepidus*, the results are applicable to other color polymorphic species which inhabit areas with contrasting substrate, especially when some degree of geographic isolation is present. Additionally, our interpretation of our data on blotched models leads us to believe that behavior and ecology of a species may play an important role in the maintenance of color or pattern polymorphism.
